# Reverse Remodeling Following Sacubitril/Valsartan Initiation in CRT-Treated HFrEF Patients: Insights from a Real-World Cohort

**DOI:** 10.3390/diagnostics16162669

**Published:** 2026-08-21

**Authors:** Oana Pătru, Dragoș Cozma, Silvia Luca, Cristina Văcărescu, Simina Crișan, Bogdan Enache, Mirela Virtosu, Constantin-Tudor Luca, Radu Vătășescu, Simona Ruxanda Drăgan

**Affiliations:** 1 Cardiology Department, “Victor Babes” University of Medicine and Pharmacy, 300041 Timisoara, Romania; oana.patru@umft.ro (O.P.); dragos.cozma@umft.ro (D.C.); silvia.luca@umft.ro (S.L.); cristina.vacarescu@umft.ro (C.V.); bogdan.enache@umft.ro (B.E.); constantin.luca@umft.ro (C.-T.L.); simona.dragan@umft.ro (S.R.D.); 2 Research Center of the Institute of Cardiovascular Diseases Timisoara, 13A Gheorghe Adam Street, 300310 Timisoara, Romania; 3 Doctoral School, “Victor Babes” University of Medicine and Pharmacy, 300041 Timisoara, Romania; daniela.cozma@umft.ro; 4 Institute of Cardiovascular Diseases Timisoara, 13A Gheorghe Adam Street, 300310 Timisoara, Romania; 5 Department of Cardiology, Faculty of Medicine, Carol Davila University of Medicine and Pharmacy, 050474 Bucharest, Romania; radu.vatasescu@umfcd.ro; 6 Department of Cardiology, Emergency Clinical Hospital of Bucharest, Calea Floreasca 8, 014461 Bucharest, Romania

**Keywords:** heart failure with reduced ejection fraction, cardiac resynchronization therapy, sacubitril/valsartan, angiotensin receptor–neprilysin inhibitor, reverse remodeling, left ventricular ejection fraction, treatment timing

## Abstract

**Background:** Sacubitril/valsartan is a cornerstone of guideline-directed medical therapy for heart failure with reduced ejection fraction (HFrEF), yet data regarding reverse remodeling after ARNI initiation in patients previously treated with cardiac resynchronization therapy (CRT) remain limited. This study evaluated echocardiographic reverse remodeling following sacubitril/valsartan initiation in a real-world cohort of CRT-treated patients and explored the association between treatment timing and remodeling response. **Methods:** This single-center retrospective pilot study included 188 patients with HFrEF treated with CRT who subsequently initiated sacubitril/valsartan. Patients were categorized into early (≤12 months after CRT, *n* = 112) and late (>12 months, *n* = 76) initiation groups. Echocardiographic parameters and functional status were assessed at baseline and at approximately 12 months. Reverse remodeling was evaluated using changes in left ventricular ejection fraction (LVEF), ventricular volumes, and clinical status. Multivariable logistic regression was used to explore factors associated with reverse remodeling (ΔLVEF ≥ 10%). **Results:** Sacubitril/valsartan therapy was associated with significant improvements in LVEF, left ventricular end-diastolic volume, left atrial volume, and NYHA functional class in both groups. The magnitude of improvement in echocardiographic parameters was similar between early and late initiation groups. In exploratory multivariable analyses, earlier ARNI initiation was associated with clinically meaningful reverse remodeling (ΔLVEF ≥ 10%) (OR 6.36, 95% CI 1.59–25.50, *p* = 0.009). SGLT2 inhibitor therapy was also associated with reverse remodeling (OR 5.76, 95% CI 1.86–17.87, *p* = 0.002), while a longer CRT-to-ARNI interval was associated with lower odds of response (OR 0.77 per year, 95% CI 0.62–0.96, *p* = 0.018). Analysis of CRT-to-ARNI interval as a continuous variable showed only a weak association with reverse remodeling, while receiver operating characteristic analysis did not identify a meaningful temporal threshold (AUC 0.497). **Conclusions:** Sacubitril/valsartan initiation after CRT was associated with significant reverse remodeling, including in patients who initiated therapy several years after CRT implantation, although the late-initiation subgroup was of limited size, and treatment intervals beyond the interquartile range (4.0–7.0 years) were sparsely represented. Absolute echocardiographic improvements were broadly similar between groups, and receiver operating characteristic analysis did not identify a discriminative temporal threshold (AUC 0.497), indicating no discriminative ability beyond chance. Exploratory multivariable analysis identified an association between earlier initiation and clinically meaningful reverse remodeling, but this finding was not supported by a clinically meaningful temporal threshold.

## 1. Introduction

Heart failure with reduced ejection fraction (HFrEF) remains a major cause of morbidity and mortality despite substantial advances in pharmacological and device-based therapies. Progressive ventricular remodeling, characterized by left ventricular dilation and impaired systolic function, represents a key determinant of disease progression and clinical outcomes. Current management strategies aim not only to improve symptoms but also to promote reverse remodeling and stabilize cardiac function [1,2].

Cardiac resynchronization therapy (CRT) is an established treatment for selected patients with HFrEF and ventricular conduction delay. By restoring ventricular synchrony, CRT improves left ventricular function, reduces ventricular volumes, and alleviates functional mitral regurgitation, leading to improved functional status and prognosis. Nevertheless, the magnitude of reverse remodeling after CRT varies considerably, and a substantial proportion of patients exhibit incomplete structural recovery despite adequate device function [3,4].

Angiotensin receptor–neprilysin inhibitors (ARNIs), particularly sacubitril/valsartan, represent a cornerstone of guideline-directed medical therapy (GDMT) for HFrEF and have been consistently associated with improved clinical outcomes and significant reverse ventricular remodeling [4]. Because pharmacological reverse remodeling targets neurohormonal activation while CRT addresses electrical dyssynchrony, the combination of these therapies may provide complementary benefits [2,5].

Observational data suggest that ARNI therapy may enhance structural and functional improvement in patients treated with CRT, including improvements in ventricular function, clinical status, and quality of life [6,7,8]. Importantly, beneficial effects have been reported even when ARNI therapy is introduced after a prolonged period following CRT implantation, suggesting that pharmacological remodeling may remain achievable in patients with long-standing device therapy [9].

Despite the increasing use of combined CRT and ARNI therapy, the optimal timing of ARNI initiation relative to device implantation remains insufficiently defined. Most available studies have focused primarily on comparisons between ARNI-treated and non-ARNI-treated patients [6,7,8,9,10,11], while relatively little attention has been given to the influence of treatment timing in CRT recipients [9]. It remains unclear whether the timing of sacubitril/valsartan initiation after CRT implantation influences the extent of reverse remodeling. In routine clinical practice, the timing of ARNI initiation is influenced not only by clinical considerations but also by treatment availability and healthcare system factors. Such real-world variability offers an opportunity to evaluate the impact of treatment timing on cardiac remodeling.

Therefore, the aim of the present study was to evaluate echocardiographic reverse remodeling following sacubitril/valsartan initiation in CRT-treated HFrEF patients and to explore the association between treatment timing and remodeling response.

## 2. Materials and Methods

### 2.1. Study Design and Population

This was a single-center retrospective pilot observational cohort study conducted at a tertiary cardiology center. The study included consecutive patients with HFrEF treated with CRT who subsequently received sacubitril/valsartan therapy during follow-up. The study protocol was approved by the institutional ethics committee and conducted in accordance with the Declaration of Helsinki. Due to the retrospective design and use of routinely collected data, no additional informed consent was required beyond standard hospital admission consent.

Baseline (T0) was defined as the time of sacubitril/valsartan initiation, while follow-up evaluation (T1) corresponded to approximately 12 months (±2 months) after therapy initiation. Eligible patients were adults with chronic HFrEF (LVEF ≤ 40%) who had previously undergone CRT implantation and subsequently initiated sacubitril/valsartan therapy. The study cohort was defined at the time of ARNI initiation (T0) and included only patients who were alive, under active follow-up, and had complete echocardiographic examinations at baseline and follow-up. As such, the study population represents a selected subgroup of CRT recipients who remained clinically stable and eligible for ARNI therapy, rather than the entire population of patients undergoing CRT implantation. Patients with missing echocardiographic data, severe primary valvular disease requiring intervention, congenital heart disease, or inadequate device function were excluded.

Patients were categorized according to the timing of sacubitril/valsartan initiation relative to CRT implantation. Early initiation was defined as ≤12 months after CRT and late initiation as >12 months. This threshold was selected a priori as a pragmatic distinction between early post-implant optimization and delayed treatment escalation in routine clinical practice, rather than a physiologically defined cutoff. The distribution of treatment timing reflected real-world clinical practice during a transitional period in heart failure management, influenced in part by the timing of national reimbursement availability for sacubitril/valsartan. As a result, many patients in the late initiation group had undergone CRT implantation several years before ARNI therapy became routinely accessible, leading to delayed initiation despite continued clinical follow-up. In contrast, patients treated more recently generally received earlier ARNI initiation following device implantation, in accordance with evolving guideline-directed medical therapy strategies. In current practice, sacubitril/valsartan is increasingly initiated prior to CRT implantation when clinically feasible, which may reduce the occurrence of similar delayed initiation patterns in future cohorts. To address the limitations of categorical classification, the CRT-to-ARNI interval was also analyzed as a continuous variable.

Clinical data were obtained from electronic medical records and included age, sex, heart failure etiology (ischemic or non-ischemic), and NYHA functional class.

Most patients were receiving GDMT according to contemporary heart failure management recommendations. Beta-blocker therapy was routinely titrated to the maximally tolerated dose whenever clinically feasible in order to maintain adequate heart rate control and ensure a high percentage of effective biventricular pacing. Background therapy before ARNI initiation included angiotensin-converting enzyme inhibitors or angiotensin receptor blockers, mineralocorticoid receptor antagonists, sodium-glucose cotransporter-2 inhibitors when indicated, and loop diuretics according to clinical status. Sacubitril/valsartan therapy was initiated during a dedicated hospital admission at baseline (T0), according to a standardized institutional protocol based on contemporary ESC and Heart Failure Association recommendations and national reimbursement regulations. The initial dose (24/26 mg or 49/51 mg twice daily) was selected according to blood pressure, renal function, serum potassium levels, and prior treatment with angiotensin-converting enzyme inhibitors or angiotensin receptor blockers. Dose titration toward the maximally tolerated dose was performed during outpatient follow-up visits at approximately 4–8-week intervals, and the tolerated maintenance dose was recorded at follow-up. 

Device-related parameters included device type, percentage of effective biventricular pacing, and baseline QRS duration. Patients were treated with CRT devices including CRT-P, CRT-D, or left-ventricular-only pacing systems with right atrial and left ventricular leads (RA–LV configuration), which were considered a CRT-P equivalent for analytical purposes. All patients had adequate device function and a high percentage of effective biventricular pacing prior to ARNI initiation, as assessed during routine device follow-up.

### 2.2. Echocardiographic Assessment

Transthoracic echocardiography was performed at baseline and follow-up using standard imaging protocols in the same echocardiography laboratory, in accordance with current ASE/EACVI recommendations, using VIVID echocardiography systems (GE Healthcare, Milwaukee, WI, USA) with a 2.5 MHz transducer.

Baseline echocardiography corresponded to the time of sacubitril/valsartan initiation, while follow-up examinations were performed approximately 12 months after therapy initiation (±2 months). Left ventricular ejection fraction (LVEF) was calculated using the biplane Simpson method. Left ventricular end-diastolic volume (LVEDV) and left atrial volume (LAV) were measured according to standard recommendations, with LAV assessed at end-systole.

Mitral regurgitation (MR) severity was evaluated using an integrative semi-quantitative approach and graded on a four-level scale (grade I–IV) according to current echocardiographic guidelines. MR improvement was defined as a reduction of at least one grade between baseline and follow-up.

Trans-mitral inflow velocities were assessed using pulsed-wave Doppler and the E/A ratio was calculated. Diastolic function parameters were analyzed only in patients in sinus rhythm; patients with permanent atrial fibrillation were excluded from analyses involving these measurements.

All measurements were performed by experienced operators.

### 2.3. Arrhythmias

Arrhythmic events were identified based on device interrogation reports and electrocardiographic documentation. Recorded arrhythmias included atrial fibrillation and ventricular tachycardia. Arrhythmic status was assessed both at baseline and at follow-up.

### 2.4. Study Endpoints

The primary endpoint of the study was the change in LVEF (ΔLVEF) between baseline and follow-up. In addition, reverse remodeling was defined a priori as an absolute increase in LVEF ≥ 5%, with a secondary exploratory analysis using a stricter threshold of ≥10%. An absolute increase in LVEF of ≥10 percentage points was considered indicative of clinically meaningful reverse remodeling, as this threshold exceeds expected measurement variability and has been commonly used in studies evaluating remodeling response in HFrEF and CRT populations [3,11].

Secondary endpoints included changes in LVEDV (ΔLVEDV), LAV, NYHA functional class, MR severity, and E/A ratio in patients with sinus rhythm, as well as arrhythmic events during follow-up. Reverse remodeling was assessed based on changes in left ventricular ejection fraction and ventricular volumes. Improvement in MR severity was defined as a reduction of at least one grade between baseline and follow-up.

### 2.5. Statistical Analysis

Statistical analysis was performed using MedCalc Statistical Software version 12.5 (MedCalc Software Ltd., Ostend, Belgium).

Continuous variables were assessed for normality using the Shapiro–Wilk test and are presented as mean ± standard deviation (SD) or median with interquartile range (IQR), as appropriate. Categorical variables are presented as counts and percentages. Within-group comparisons between baseline and follow-up were performed using paired *t*-tests or Wilcoxon signed-rank tests for continuous variables, and the McNemar test for categorical variables such as NYHA functional class and MR grade. Between-group comparisons (early vs. late ARNI initiation) were performed using independent-samples *t*-tests or Mann–Whitney U tests for continuous variables and chi-square or Fisher’s exact tests for categorical variables, as appropriate. Changes between baseline and follow-up were calculated as delta values (Δ), defined as follow-up minus baseline, for key echocardiographic parameters (e.g., ΔLVEF, ΔLVEDV, ΔLAV), and were compared between groups.

Multivariable logistic regression analysis was performed to explore factors associated with reverse remodeling. Reverse remodeling was defined as a binary variable based on changes in LVEF. Responders were initially defined as patients with ΔLVEF ≥ 5%. Because a large proportion of patients exceeded this threshold, a secondary analysis was performed using a stricter definition of response (ΔLVEF ≥ 10%). All variables were entered simultaneously into the model and included age, sex, cardiomyopathy etiology, CRT-to-ARNI interval, ARNI daily dose, SGLT2 inhibitor therapy, baseline NYHA functional class, baseline QRS duration, and early versus late ARNI initiation group. The interval between CRT implantation and ARNI initiation was analyzed as a continuous variable. Associations between CRT-to-ARNI interval and echocardiographic reverse remodeling (ΔLVEF and ΔLVEDV) were examined using Pearson or Spearman correlation coefficients, as appropriate. Exploratory receiver operating characteristic (ROC) analysis was performed to explore whether the CRT-to-ARNI interval could discriminate patients with meaningful reverse remodeling, defined as an improvement in LVEF ≥ 10%. The area under the ROC curve (AUC) and the optimal cutoff value according to the Youden index were calculated.

All statistical tests were two-sided, and a *p* value < 0.05 was considered statistically significant.

## 3. Results

### 3.1. Study Population and Baseline Characteristics

A total of 188 patients with HFrEF treated with CRT and subsequently initiated on sacubitril/valsartan were included in the study. Among these, 112 patients were classified in the early ARNI initiation group and 76 in the late initiation group. At baseline, most patients were in NYHA functional class II or III.

Baseline clinical, device-related, and echocardiographic characteristics are summarized in Table 1.

The interval between CRT implantation and ARNI initiation was minimal in the early initiation group, with a median duration of 1 month (IQR 0–3 months). In contrast, patients in the late initiation group initiated ARNI therapy after a median interval of 5.5 years (IQR 4.0–7.0 years, range 1–12 years).

Baseline GDMT before ARNI initiation is summarized in Table 2.

Overall, the two groups were comparable with respect to age, sex, cardiomyopathy etiology, device type, and percentage of effective biventricular pacing. However, several clinically relevant baseline differences were present, including LVEF, QRS duration, NYHA functional class, and MR severity.

Patients in the early ARNI initiation group had slightly lower baseline left ventricular ejection fraction and longer QRS duration compared with the late initiation group. The distribution of NYHA functional class differed modestly, with a higher proportion of patients in NYHA class II in the late ARNI group. Baseline MR severity also differed between groups.

The prevalence of device-detected arrhythmias at baseline was similar between groups, affecting approximately one third of patients.

### 3.2. Treatment Characteristics

Sacubitril/valsartan therapy was initiated and titrated according to routine clinical practice. The distribution of achieved maintenance ARNI dose at follow-up was comparable between the early and late initiation groups (Table 3, *p* = 0.219), with most patients receiving 200 mg/day, followed by 100 mg/day and 400 mg/day.

### 3.3. Echocardiographic and Functional Remodeling

Changes in echocardiographic parameters and functional status are summarized in Table 4.

**Figure 1:**
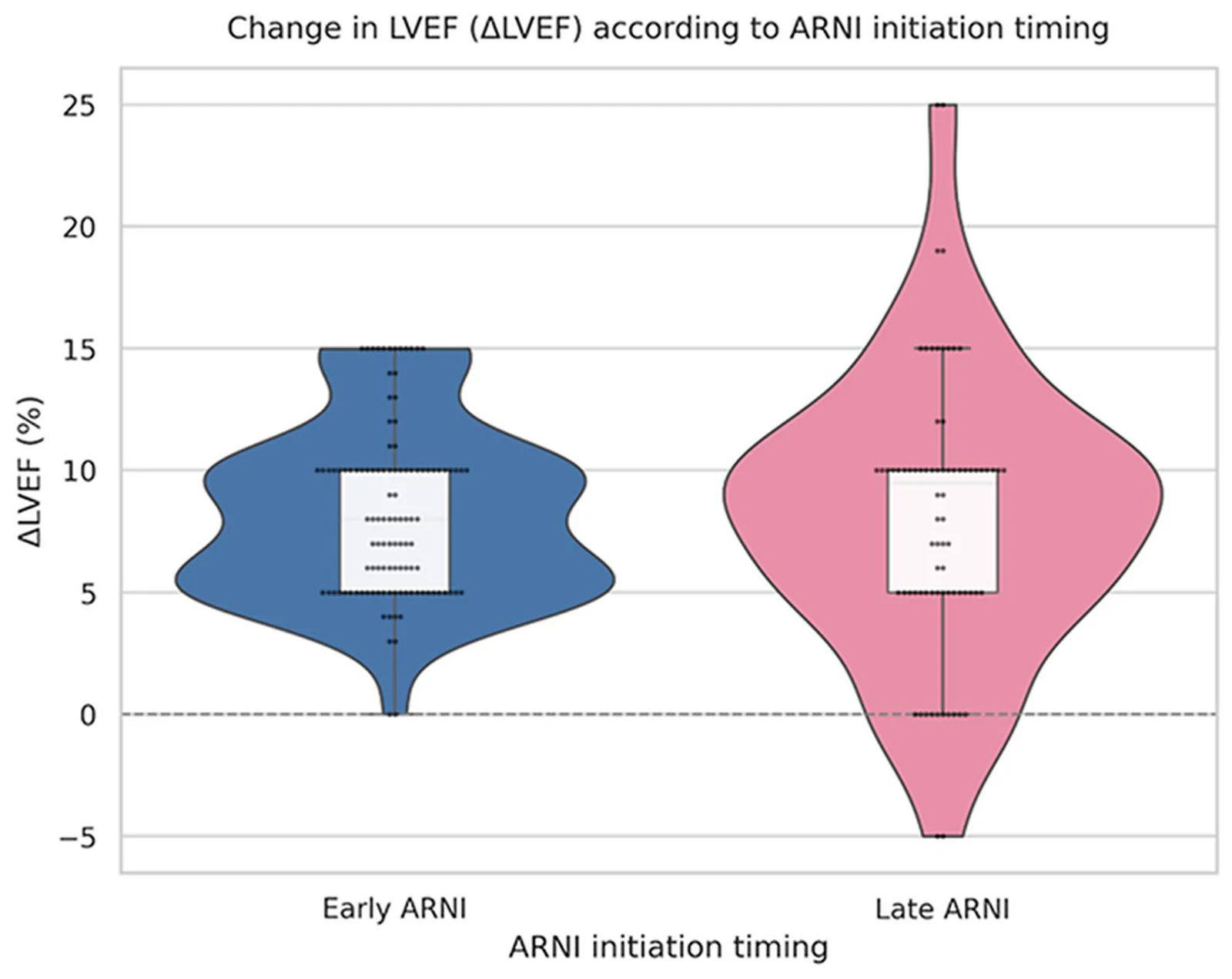
Change in LVEF (ΔLVEF) according to timing of sacubitril/valsartan initiation. Violin plots with embedded boxplots show the distribution, median, and IQR of ΔLVEF values. The individual black dots represent individual patient observations, overlaid to illustrate the distribution of ΔLVEF values within each group. No significant between-group difference was observed (*p* = 0.826).

Both groups demonstrated significant improvement in left ventricular systolic function over follow-up, with comparable increases in LVEF. The distribution of ΔLVEF according to ARNI initiation timing is illustrated in Figure 1 and showed a similar overall pattern of functional improvement between groups.

Markers of structural reverse remodeling improved in both cohorts. LVEDV and LAV decreased significantly during follow-up, with no statistically significant differences between groups in the magnitude of change.

MR severity decreased in both groups, with a reduction in the proportion of patients with moderate-to-severe regurgitation and no cases of grade IV regurgitation at follow-up. Improvement of at least one MR grade was more frequently observed in the early ARNI initiation group.

Trans-mitral inflow parameters showed only modest changes. In patients in sinus rhythm, the E/A ratio did not change significantly in either group.

NYHA functional class improved in both groups, with a shift toward lower symptom burden at follow-up. The magnitude of improvement was similar between groups.

### 3.4. Comparison Between Early and Late ARNI Initiation

Comparisons of changes between baseline and follow-up are summarized in Table 5.

Overall, the magnitude of improvement was similar between the early and late ARNI initiation groups. Changes in LVEF, LVEDV, and LAV did not differ significantly between groups. Changes in NYHA functional class were comparable between groups (*p* = 0.058). Similarly, changes in trans-mitral E/A ratio were modest and did not differ between groups.

Improvement in MR by at least one grade was observed more frequently in the early ARNI initiation group than in the late initiation group (67.8% vs. 52.6%, *p* = 0.035).

### 3.5. Exploratory Predictors of Reverse Remodeling

Initially, responders were defined as patients with an absolute increase in LVEF of at least 5 percentage points (ΔLVEF ≥ 5%). Using this definition, 168 of 188 patients (89.4%) were classified as responders. Given this high response rate, the discriminative capacity of the model was limited.

In the corresponding multivariable logistic regression analysis, non-ischemic cardiomyopathy etiology was independently associated with ΔLVEF ≥ 5% (OR 3.18, 95% CI 1.33–8.32, *p* = 0.013), while longer CRT-to-ARNI interval was associated with a lower likelihood of response (OR 0.78 per year, 95% CI 0.66–0.92, *p* = 0.005). When ARNI dose was analyzed categorically using 100 mg/day as the reference, both 200 mg/day (OR 2.97, 95% CI 1.07–7.53, *p* = 0.026) and 400 mg/day (OR 4.28, 95% CI 2.22–7.09, *p* < 0.001) were associated with higher odds of response. No significant associations were observed for age, sex, SGLT2 inhibitor therapy, baseline NYHA class, baseline QRS duration, or ARNI initiation timing.

Because the ≥5% threshold classified a large proportion of patients as responders and provided limited discriminatory capacity, a stricter definition of clinically meaningful reverse remodeling (ΔLVEF ≥ 10%) was additionally explored. Using this definition, 86 of 188 patients (45.7%) were classified as responders. 

In exploratory multivariable analysis, early ARNI initiation was associated with a higher likelihood of response (OR 6.36, 95% CI 1.59–25.50, *p* = 0.009). SGLT2 inhibitor therapy was also associated with clinically meaningful reverse remodeling (OR 5.76, 95% CI 1.86–17.87, *p* = 0.002), while longer CRT-to-ARNI interval remained associated with a lower probability of achieving ΔLVEF ≥ 10% (OR 0.77 per year, 95% CI 0.62–0.96, *p* = 0.018). No significant associations were observed for age, sex, cardiomyopathy etiology, baseline NYHA class, baseline QRS duration, or ARNI dose.

These exploratory findings suggest that the stricter ΔLVEF ≥ 10% definition may better discriminate clinically relevant remodeling patterns within this cohort.

The relationship between achieved maintenance ARNI dose and reverse remodeling was further explored. When response was defined as ΔLVEF ≥ 5%, responders were more likely to receive higher maintenance doses, whereas non-responders were predominantly treated with 100 mg/day (*p* = 0.0006). In contrast, when using the stricter definition of ΔLVEF ≥ 10%, the distribution of ARNI dose was less clearly differentiated between responders and non-responders (*p* = 0.076) (Table 6).

**Figure 2:**
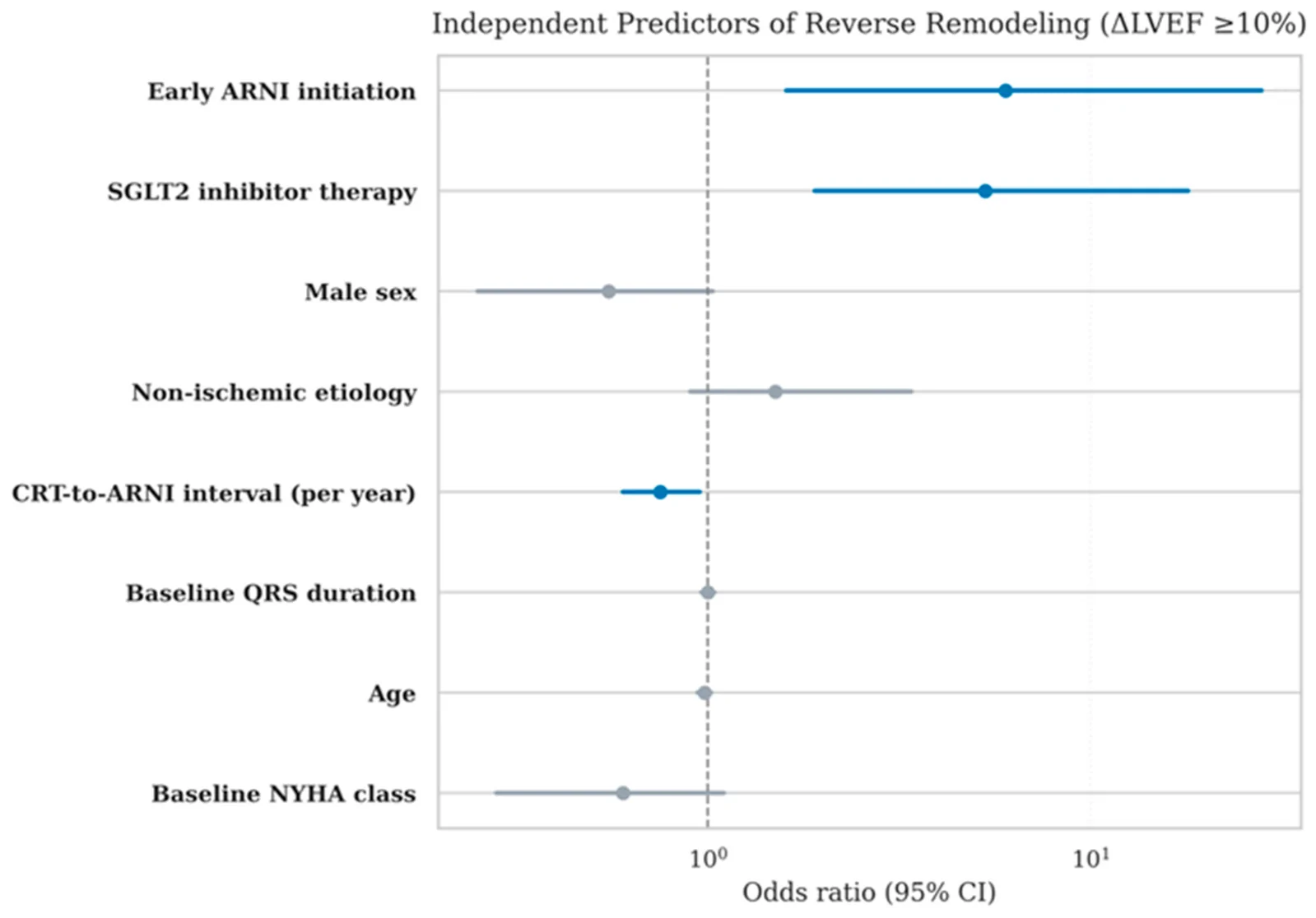
Exploratory predictors of reverse remodeling (ΔLVEF ≥ 10%) derived from multivariable logistic regression. ORs and 95% CIs are shown on a logarithmic scale. Blue indicates statistically significant associations (95% CI not crossing the null value), whereas gray indicates non-significant associations. Dots represent the point estimates of the ORs, and horizontal lines represent the corresponding 95% CIs. The vertical dashed line indicates an OR of 1.0, representing the null association.

Independent predictors of reverse remodeling are illustrated in Figure 2.

### 3.6. Arrhythmic Events

The baseline prevalence of any device-detected arrhythmia was similar between groups (Table 1). 

At baseline, atrial fibrillation (AF) was present in 28/112 patients (25.0%) in the early group and 24/76 (31.6%) in the late group (*p* = 0.410). At follow-up, AF was present in 12/112 (10.7%) and 6/76 (7.9%), respectively (*p* = 0.695).

Ventricular tachycardia (VT) was documented in 8/112 patients (7.1%) in the early group and 4/76 (5.3%) in the late group at baseline (*p* = 0.831), decreasing to 2/112 (1.8%) and 0/76 (0.0%) at follow-up (*p* = 0.655).

### 3.7. Timing of ARNI Initiation and Reverse Remodeling

The interval between CRT implantation and ARNI initiation was analyzed as a continuous variable. Longer CRT-to-ARNI duration was weakly associated with smaller improvements in left ventricular ejection fraction (*r* = −0.149, *p* = 0.039).

**Figure 3:**
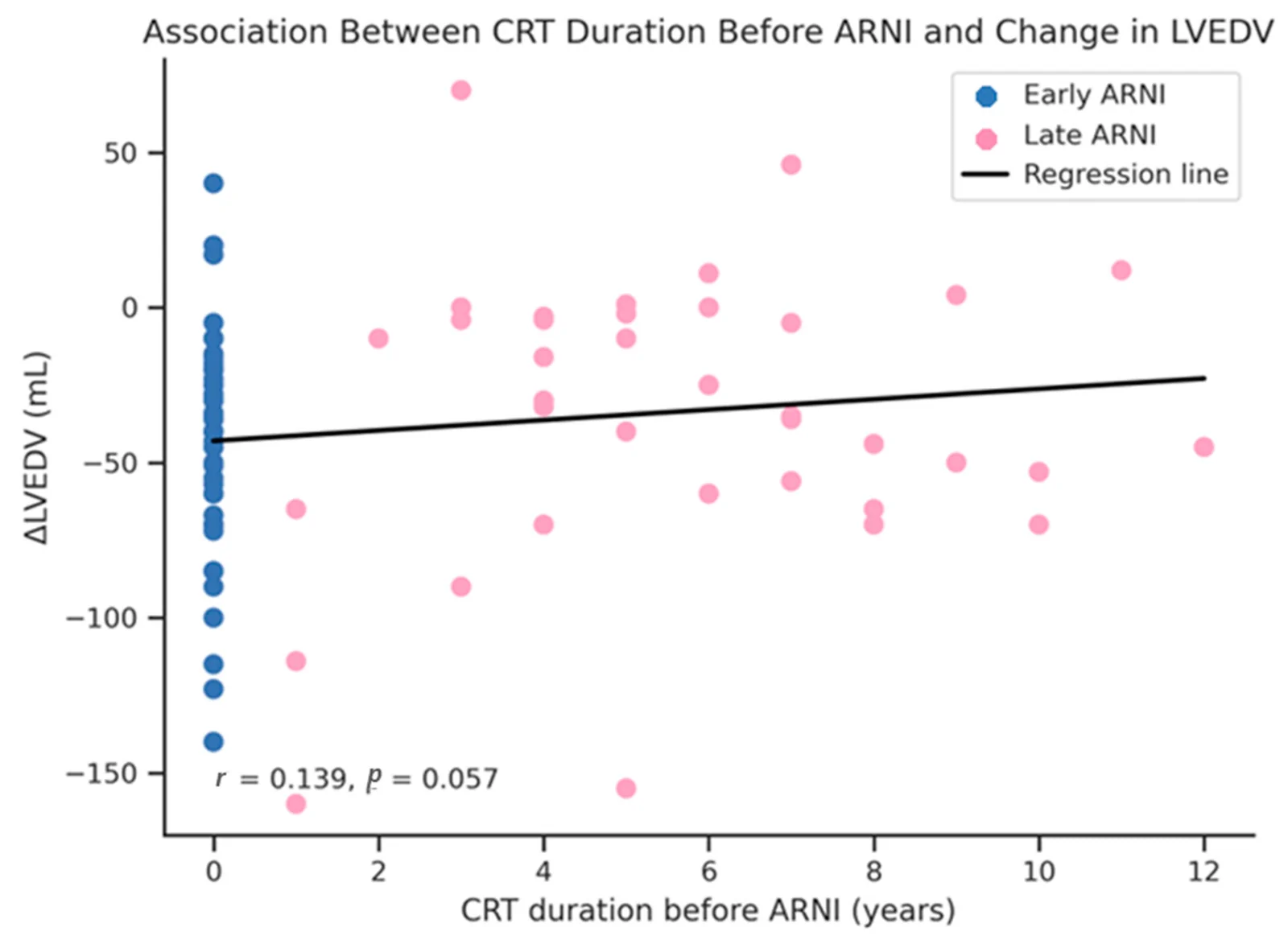
Relationship between CRT-to-ARNI interval and change in LVEDV (ΔLVEDV). Scatter plot with linear regression line illustrating the association between treatment timing and LV reverse remodeling. Blue dots represent patients in the early ARNI initiation group, pink dots represent patients in the late ARNI initiation group, and the black line represents the linear regression line. The correlation coefficient and corresponding *p*-value are shown in the plot (*r* = 0.139, *p* = 0.057).

No significant association was observed between CRT-to-ARNI interval and change in LVEDV (*r* = 0.139, *p* = 0.057) (Figure 3).

**Figure 4:**
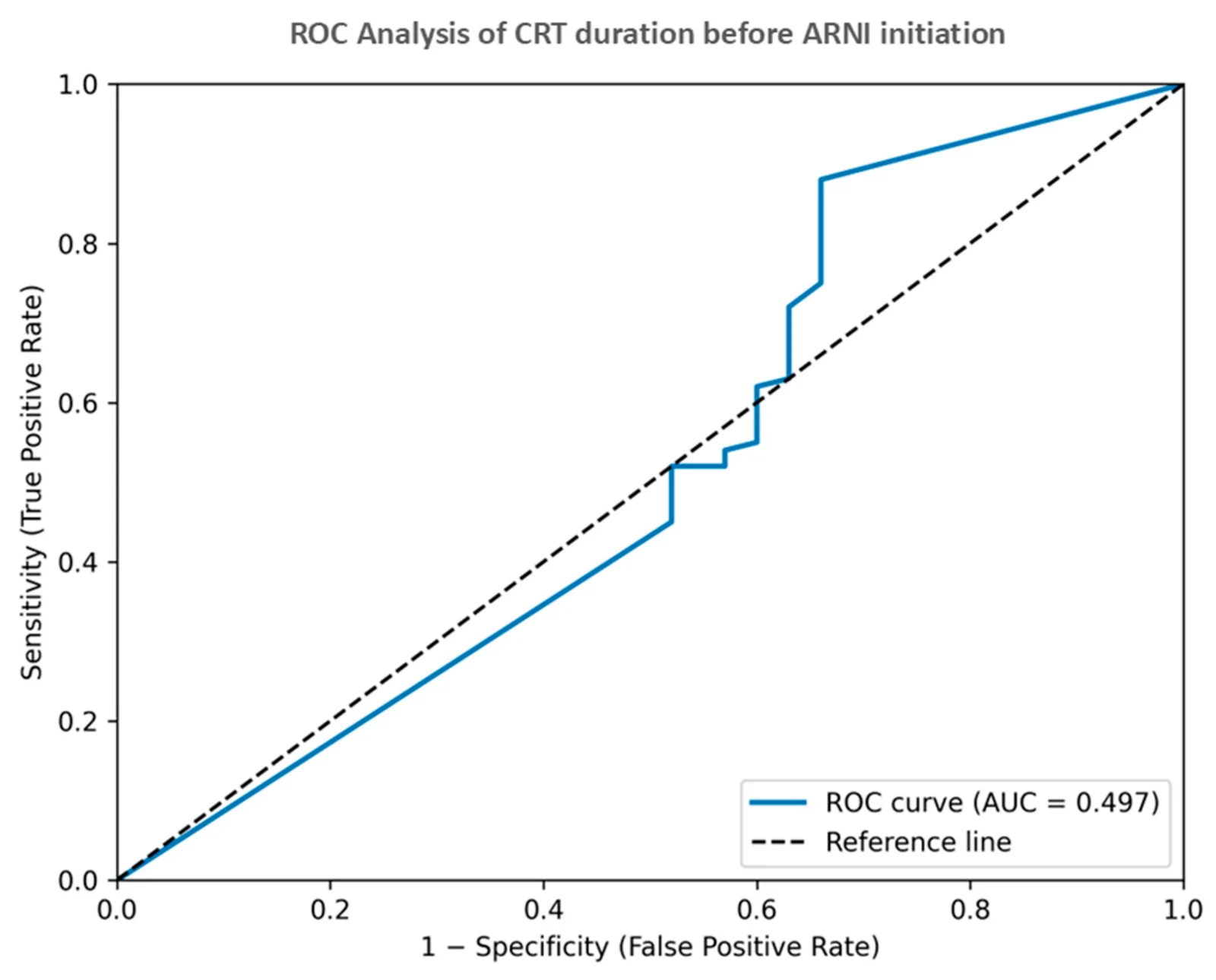
Receiver operating characteristic (ROC) curve of the CRT-to-ARNI interval for predicting clinically meaningful reverse remodeling (ΔLVEF ≥ 10%). The area under the curve (AUC) was 0.497 (95% CI, 0.424–0.573), indicating no discriminative ability beyond chance. The maximum Youden index was 0.069 at a CRT-to-ARNI interval of 1.0 year (sensitivity 44.2%, specificity 62.7%), indicating that no clinically meaningful temporal threshold could be identified.

Exploratory receiver operating characteristic analysis did not identify a clinically meaningful temporal threshold for predicting reverse remodeling (ΔLVEF ≥ 10%) (AUC 0.497; 95% CI 0.424–0.573) (Figure 4). The maximum Youden index was 0.069, corresponding to a CRT-to-ARNI interval of 1.0 year (sensitivity 44.2%, specificity 62.7%), indicating no useful discriminatory threshold within this cohort.

## 4. Discussion

### 4.1. Principal Findings

The present study evaluated the association between the timing of sacubitril/valsartan initiation and echocardiographic reverse remodeling in patients with HFrEF treated with CRT.

Sacubitril/valsartan therapy was associated with significant reverse remodeling in this population, as reflected by improvements in LVEF and reductions in ventricular and atrial volumes during follow-up. These beneficial effects were observed in both early and late ARNI initiation groups.

Absolute improvements in LVEF, LVEDV, and LAV were broadly similar between the early and late initiation groups. Exploratory multivariable analyses suggested a possible association between earlier sacubitril/valsartan initiation and a higher likelihood of achieving clinically meaningful reverse remodeling, defined as an absolute increase in LVEF ≥ 10%. This observation should be interpreted cautiously in light of baseline differences between the study groups, the weak continuous association between the CRT-to-ARNI interval and remodeling, the absence of a meaningful temporal threshold, and the possibility of differing remodeling trajectories. Importantly, clinically relevant improvements were also observed in patients who initiated ARNI therapy several years after CRT implantation, although this subgroup was limited in size and the longer intervals within it were sparsely represented.

Improvements in MR severity and functional status were observed in both groups. Although arrhythmic events were numerically less frequent at follow-up in both groups, no significant between-group differences were observed, and these findings should be interpreted as descriptive.

In addition to the categorical comparison, analysis of the CRT-to-ARNI interval as a continuous variable showed a weak association between longer delays and smaller improvements in reverse remodeling. Exploratory receiver operating characteristic analysis did not identify a meaningful temporal threshold, suggesting that no specific cut-off for ARNI initiation could be identified within the present cohort.

### 4.2. Early ARNI Initiation and Reverse Remodeling

The exploratory association between earlier initiation of sacubitril/valsartan and a higher likelihood of clinically meaningful reverse remodeling may be consistent with the hypothesis that timely neurohormonal optimization after CRT implantation could facilitate the structural response to resynchronization therapy [12,13,14]. CRT improves ventricular synchrony and mechanical efficiency, thereby creating favorable conditions for reverse remodeling, while sacubitril/valsartan further modulates neurohormonal activation and reduces myocardial wall stress [4,15].

The complementary effects of electrical resynchronization and neurohormonal blockade provide a biologically plausible rationale for the observed findings. However, because the present study was observational and continuous measures of remodeling were broadly similar between groups, these results should not be interpreted as evidence that earlier initiation is superior to delayed treatment.

Importantly, clinically meaningful structural and functional improvements were also observed in patients who initiated sacubitril/valsartan several years after CRT implantation. Although exploratory multivariable analyses suggested a possible association between earlier ARNI initiation and clinically meaningful reverse remodeling, this observation should be interpreted cautiously because continuous measures of remodeling were broadly similar between groups and no meaningful temporal threshold was identified. Moreover, the continuous association between the CRT-to-ARNI interval and reverse remodeling was weak, and receiver operating characteristic analysis did not identify a meaningful temporal threshold. Taken together, these findings suggest that the concept of a temporal “window of myocardial plasticity” remains speculative and was not directly demonstrated in the present study.

### 4.3. Clinical Impact of Late ARNI Initiation

An important observation of our study was that clinically meaningful improvements were also observed in patients who initiated sacubitril/valsartan several years after CRT implantation. Despite a longer duration of established ventricular dysfunction prior to ARNI initiation, patients in the late initiation group showed improvements in left ventricular function, ventricular volumes, and functional status.

Patients in the late initiation group likely represent a selected population of long-term CRT survivors who remained clinically stable and eligible for ARNI therapy several years after device implantation. Consequently, survivor bias and differences in baseline remodeling trajectory may have influenced the observed response patterns. In clinical practice, many CRT recipients in this cohort remained on conventional renin–angiotensin system blockade for prolonged periods before transitioning to ARNI therapy, particularly in healthcare systems where treatment availability was influenced by reimbursement policies.

These observations describe reverse remodeling and functional improvement following ARNI initiation in a selected population of long-term CRT survivors. Given the observational design and the limited size and distribution of the late initiation group, they should not be interpreted as evidence of a specific clinical benefit associated with delayed ARNI initiation.

### 4.4. Relation to Previous Studies

Previous studies have consistently demonstrated that sacubitril/valsartan therapy is associated with improvements in ventricular function and reverse remodeling in patients with HFrEF [12,16,17,18]. Similar beneficial effects have been reported in patients treated with CRT [19,20] supporting the concept that ARNI therapy may complement device-based treatment.

However, most available studies have focused on comparisons between ARNI-treated and non-ARNI-treated patients, and data regarding the influence of treatment timing in CRT recipients remain limited. Previous CRT studies have described substantial reverse remodeling and heterogeneous patterns of response across different patient populations and pacing strategies [21,22]. Existing observational studies, often involving relatively small and heterogeneous populations, have suggested that sacubitril/valsartan may improve echocardiographic parameters and clinical outcomes in CRT-treated patients, including those with persistent ventricular dysfunction despite long-standing device therapy [7,8,11,14]. Improvements in functional capacity and quality of life have also been described following delayed ARNI initiation [9].

The present study extends these observations by evaluating reverse remodeling following sacubitril/valsartan initiation in a real-world cohort of CRT-treated patients, including patients who initiated ARNI therapy more than 12 months after CRT. Exploratory analyses of treatment timing did not identify a clinically meaningful temporal threshold, and these findings should therefore be considered hypothesis-generating.

### 4.5. Interpretation in the Context of Current Clinical Practice

The findings of the present study should be interpreted within the real-world context represented by this cohort rather than as evidence supporting a specific treatment strategy. Current HF guidelines [1] recommend early optimization of GDMT, including angiotensin receptor–neprilysin inhibitors, prior to consideration of device-based therapies such as CRT. However, the present cohort reflects an earlier period in which many patients underwent CRT implantation before full optimization of medical therapy because of evolving treatment paradigms, reimbursement policies, and limited access to ARNI therapy.

Within this observational cohort, improvements in echocardiographic parameters and functional status were observed following sacubitril/valsartan initiation across different intervals after CRT implantation. However, given the limited sample size, the imbalance in treatment timing, the observational design, baseline differences between groups, and the sparse representation of longer CRT-to-ARNI intervals, these findings should not be interpreted as evidence for a preferred timing of ARNI initiation or as a basis for clinical treatment recommendations.

### 4.6. Study Limitations

Several limitations of the present study should be acknowledged. First, this was a single-center retrospective pilot observational study, which may limit generalizability and introduces the possibility of selection bias. Second, treatment allocation was not randomized, and the timing of sacubitril/valsartan initiation reflected real-world clinical practice, including the influence of reimbursement availability. In addition, the timing of ARNI initiation was influenced by real-world clinical factors, including historical treatment availability, reimbursement policies, evolving heart failure management strategies, and potentially physician-related treatment decisions. Consequently, indication bias cannot be fully excluded, and treatment timing may partly reflect differences in clinical trajectory rather than an isolated therapeutic effect. Detailed longitudinal data regarding the type of prior renin–angiotensin system blockade (ACE inhibitor versus ARB), dose intensity, and duration of therapy before ARNI initiation were not systematically available. Therefore, differences in cumulative exposure to conventional neurohormonal blockade before ARNI initiation cannot be fully excluded and may have contributed to the observed remodeling patterns, particularly in patients who initiated ARNI therapy later after CRT implantation. Although baseline characteristics were broadly comparable, residual confounding cannot be excluded. Third, baseline evaluation was defined at the time of ARNI initiation rather than at the time of CRT implantation. Therefore, the present study does not assess the early response to CRT itself but rather remodeling observed following ARNI initiation in previously resynchronized patients. Longitudinal echocardiographic data between CRT implantation and ARNI initiation were not systematically available, precluding a formal assessment of pre-ARNI remodeling trajectories. Although routine clinical follow-up indicated that CRT-related remodeling had likely occurred prior to ARNI initiation in many cases in the late ARNI group, this could not be formally quantified and remains a limitation of the present study. Thus, differences in remodeling reserve and underlying disease trajectory between groups cannot be fully excluded. In addition, although the multivariable logistic regression models included only clinically relevant covariates, the relatively limited number of events in relation to the number of variables entered into the models may have affected model stability. Therefore, these exploratory analyses should be interpreted cautiously and considered hypothesis-generating, requiring confirmation in larger prospective cohorts. Moreover, within the late initiation group, CRT-to-ARNI intervals were concentrated around the interquartile range (4.0–7.0 years), with limited representation at more extended intervals; conclusions regarding reverse remodeling at very long CRT-to-ARNI intervals therefore remain particularly speculative. Consistent with this, receiver operating characteristic analysis of the CRT-to-ARNI interval yielded an AUC of 0.497, statistically equivalent to chance, underscoring the absence of a reliable temporal threshold.

Fourth, the assessment of MR severity was based on semi-quantitative grading and may be subject to interobserver variability. Fifth, the potential influence of concomitant SGLT2 inhibitor therapy cannot be fully excluded. Sixth, natriuretic peptide levels were not systematically available and were not included in the analysis.

In addition, the association between achieved ARNI dose and reverse remodeling was not consistent across response definitions. While higher doses were associated with response when defined as ΔLVEF ≥ 5%, this relationship was not observed when using the stricter ≥ 10% threshold. This suggests that the dose–response relationship may be limited to more modest degrees of remodeling or may reflect residual confounding and should therefore be interpreted with caution.

Finally, the late initiation group inherently represents a selected population of long-term CRT survivors who remained clinically stable and eligible for ARNI therapy several years after device implantation. Accordingly, survivor bias and differences in baseline remodeling trajectory cannot be excluded and may have influenced both baseline phenotype and reverse remodeling potential.

### 4.7. Future Directions

Further prospective research is needed to better characterize the relationship between the timing of sacubitril/valsartan initiation and reverse remodeling in CRT-treated patients. In particular, larger prospective studies with more balanced representation across treatment intervals are needed to determine whether the timing of ARNI initiation is associated with differences in clinically meaningful reverse remodeling and long-term clinical outcomes.

Given the absence of a clear temporal threshold in the present study, future investigations should explore the relationship between the CRT-to-ARNI interval and reverse remodeling using continuous modeling approaches rather than predefined cut-offs.

In addition, studies incorporating serial echocardiographic assessment, systematic biomarker evaluation, and more detailed characterization of diastolic function may provide further insight into the mechanisms underlying reverse remodeling following ARNI initiation in CRT-treated patients. The interaction between ARNI therapy and other components of contemporary GDMT, including sodium–glucose cotransporter-2 inhibitors, also warrants further investigation.

## 5. Conclusions

In patients with HFrEF treated with CRT, sacubitril/valsartan initiation was associated with significant reverse remodeling and improvement in functional status, including among patients who initiated therapy several years after CRT implantation. Longer CRT-to-ARNI intervals were represented by a smaller and unevenly distributed subgroup, limiting interpretation of remodeling patterns at extended intervals.

Absolute echocardiographic improvements were broadly similar between the early and late initiation groups. Exploratory multivariable analyses identified an association between earlier ARNI initiation and a higher likelihood of achieving clinically meaningful reverse remodeling (ΔLVEF ≥ 10%); however, this association was observed in a threshold-based analysis, while continuous analyses and ROC assessment did not identify a clinically meaningful temporal threshold.

Overall, the findings describe reverse remodeling observed following sacubitril/valsartan initiation across different CRT-to-ARNI intervals. Further prospective studies with larger and more balanced cohorts are needed to determine whether treatment timing is associated with differences in reverse remodeling and long-term outcomes.

## Figures and Tables

**Table 1:** Baseline clinical, device-related, and echocardiographic characteristics of patients stratified according to timing of sacubitril/valsartan initiation (early vs. late ARNI). Continuous variables are presented as mean ± SD and categorical variables as counts and percentages.

Variable	Early ARNI (*n* = 112)	Late ARNI (*n* = 76)	*p*-Value
Age (years)	68.23 ± 8.43	67.58 ± 10.99	0.662
Sex (%)	Male: 68 (60.7%)	Male: 52 (68.4%)	0.355
	Female: 44 (39.3%)	Female: 24 (31.6%)	
Etiology of cardiomyopathy (%)	Ischemic: 36 (32.1%)	Ischemic: 24 (31.6%)	1
	Non-ischemic: 76 (67.9%)	Non-ischemic: 52 (68.4%)	
CRT duration before ARNI	1 month [IQR 0–3 months]	5.5 years [IQR 4.0–7.0 years]	<0.001
Pacing percentage (%)	99.31 ± 0.67	99.18 ± 2.65	0.677
Device Type	CRT-P: 68 (60.7%)	CRT-P: 44 (57.9%)	0.863
	CRT-D: 44 (39.3%)	CRT-D: 32 (42.1%)	
QRS duration (msec)	130.91 ± 8.09	126.61 ± 11.39	0.005
LVEF (%)	32.27 ± 5.68	35.08 ± 6.96	0.004
LVEDV (mL)	212.8 ± 58.2	211.8 ± 86.9	0.929
LAV (mL)	91.77 ± 30.74	92.63 ± 30.43	0.849
E/A ratio *	1.18 ± 0.79	1.12 ± 0.64	0.506
MR Grade	Grade I: 4 (3.6%)	Grade I: 12 (15.8%)	0.007
	Grade II: 46 (41.1%)	Grade II: 36 (47.4%)	
	Grade III: 54 (48.2%)	Grade III: 26 (34.2%)	
	Grade IV: 8 (7.1%)	Grade IV: 2 (2.6%)	
NYHA class	Class II: 38 (33.9%)	Class II: 42 (55.3%)	0.006
	Class III: 73 (65.2%)	Class III: 34 (44.7%)	
	Class IV: 1 (0.9%)	Class IV: 0 (0.0%)	
Any device-detected arrhythmia at baseline (%)	34 (30.4%)	26 (34.2%)	0.691

* E/A ratio was evaluated only in patients in sinus rhythm; patients with permanent atrial fibrillation were excluded from analyses involving this parameter.

**Table 2:** Baseline GDMT before ARNI initiation.

Medication	Early ARNI (*n* = 112)	Late ARNI (*n* = 76)	*p*-Value
ACEi/ARB	98 (87.5%)	66 (86.8%)	1.000
Beta-blocker	112 (100%)	76 (100%)	-
MRA	105 (93.8%)	74 (97.4%)	0.428
SGLT2 inhibitor	100 (89.3%)	60 (78.9%)	0.061
Loop diuretic	112 (100%)	76 (100%)	-

Beta-blocker therapy was routinely used and titrated whenever clinically feasible in order to maximize effective biventricular pacing.

**Table 3:** Distribution of achieved maintenance ARNI dose at follow-up according to timing of initiation.

Achieved Maintenance ARNI Dose, *n* (%)	Early (*n* = 112)	Late (*n* = 76)	*p*-Value *
100 mg/day	32 (28.6%)	30 (39.5%)	
200 mg/day	64 (57.1%)	34 (44.7%)	
400 mg/day	16 (14.3%)	12 (15.8%)	0.219

* *p*-value derived from chi-square test comparing overall dose distribution between groups.

**Table 4:** Echocardiographic and Clinical Changes Between Baseline and Follow-up.

Parameter	Early T0 (*n* = 112)	Early T1	*p*-Value	Late T0 (*n* = 76)	Late T1	*p*-Value
LVEF (%)	32.3 ± 5.7	40.5 ± 5.7	<0.001	35.1 ± 7.0	43.2 ± 8.4	<0.001
LVEDV (mL)	212.8 ± 58.2	170.3 ± 52.7	<0.001	211.8 ± 86.9	177.6 ± 74.7	<0.001
LAV (mL)	91.8 ± 30.7	76.4 ± 35.6	<0.001	92.6 ± 30.4	80.1 ± 27.7	<0.001
E/A ratio *	1.18 ± 0.79	1.06 ± 0.22	0.113	1.12 ± 0.64	1.4 ± 0.27	0.347
NYHA I	0 (0.0%)	48 (42.9%)		0 (0.0%)	10 (13%)	
NYHA II	38 (33.9%)	64 (57.1%)		42 (55.3%)	64 (84%)	
NYHA III	73 (65.2%)	0 (0.0%)		34 (44.7%)	2 (3%)	
NYHA IV	1 (0.9%)	0 (0.0%)		0 (0.0%)	0 (0%)	0.058
MR Grade I	4 (3.6%)	32 (28.6%)		12 (15.8%)	32 (42.1%)	
MR Grade II	46 (41.1%)	64 (57.1%)		36 (47.4%)	36 (47.4%)	
MR Grade III	54 (48.2%)	16 (14.3%)		26 (34.2%)	8 (10.5%)	
MR Grade IV	8 (7.1%)	0 (0.0%)		2 (2.6%)	0 (0.0%)	0.039

* E/A ratio calculated only in patients in sinus rhythm.

**Table 5:** Comparison of Changes Between Early and Late ARNI Initiation Groups.

Parameter	Early ARNI (*n* = 112)	Late ARNI (*n* = 76)	*p*-Value
ΔLVEF (%)	+8.3 ± 3.5	+8.10 ± 5.7	0.826
ΔLVEDV (mL)	−42.5 ± 33.7	−34.21 ± 46.33	0.183
ΔLAV (mL)	−15.3 ± 25.2	−12.52 ± 26.46	0.467
ΔNYHA class	−1.1 ± 0.35	−0.97 ± 0.49	0.058
ΔE/A ratio *	+0.12 ± 0.35	+0.075 ± 0.35	0.347
MR improvement ≥ 1 grade	76/112 (67.8%)	40/76 (52.6%)	0.035
Moderate-to-severe MR reduction, baseline → follow-up	55.3% → 14.3%	36.8% → 10.5%	-

* E/A ratio calculated only in patients in sinus rhythm. Arrows (→) indicate the change in the proportion of patients with moderate-to-severe mitral regurgitation from baseline to follow-up.

**Table 6:** Distribution of achieved maintenance ARNI dose according to response status.

Achieved Maintenance ARNI Dose, *n* (%)	Non-Responders (ΔLVEF < 5%, *n* = 20)	Responders (ΔLVEF ≥ 5%, *n* = 168)	Non-Responders (ΔLVEF < 10%, *n* = 102)	Responders (ΔLVEF ≥ 10%, *n* = 86)
100 mg/day	14 (70.0%)	48 (28.6%)	38 (37.3%)	24 (27.9%)
200 mg/day	6 (30.0%)	92 (54.8%)	54 (52.9%)	44 (51.2%)
400 mg/day	0 (0.0%)	28 (16.7%)	10 (9.8%)	18 (20.9%)
*p*-value *	0.0006		0.076	

* *p*-values derived from chi-square test for association between ARNI dose and response status within each response definition.

## Data Availability

The original contributions presented in this study are included in the article. Further inquiries can be directed to the corresponding author.

## Abbreviations

The following abbreviations are used in this manuscript:

ARNI	Angiotensin receptor–neprilysin inhibitor
CI	Confidence interval
CRT	Cardiac resynchronization therapy
CRT-D	Cardiac resynchronization therapy with defibrillator
CRT-P	Cardiac resynchronization therapy with pacemaker
DCM	Dilated cardiomyopathy
E/A ratio	Ratio of early (E) to late (A) diastolic trans-mitral flow velocities
ESC	European Society of Cardiology
GDMT	Guideline-directed medical therapy
HF	Heart failure
HFrEF	Heart failure with reduced ejection fraction
IQR	Interquartile range
LAV	Left atrial volume
LVEF	Left ventricular ejection fraction
LVEDV	Left ventricular end-diastolic volume
MR	Mitral regurgitation
NYHA	New York Heart Association
OR	Odds ratio
ROC	Receiver operating characteristic
SD	Standard deviation
SGLT2 inhibitor	Sodium–glucose cotransporter-2 inhibitor
